# Measuring fidelity, feasibility, costs: an implementation evaluation of a cluster-controlled trial of group antenatal care in rural Nepal

**DOI:** 10.1186/s12978-019-0840-4

**Published:** 2020-01-17

**Authors:** Alex Harsha Bangura, Isha Nirola, Poshan Thapa, David Citrin, Bishal Belbase, Bhawana Bogati, Nirmala B.K., Sonu Khadka, Lal Kunwar, Scott Halliday, Nandini Choudhury, Ryan Schwarz, Mukesh Adhikari, S. P. Kalaunee, Sharon Rising, Duncan Maru, Sheela Maru

**Affiliations:** 1 Lakewood Health System, Staples, MN USA; 2 Harvard T.H, Chan School of Public Health, Boston, MA USA; 3 University of New South Wales, School of Public Health and Community Medicine, Sydney, NSW Australia; 4 Nyaya Health Nepal, Kathmandu, Nepal; 5 Department of Anthropology, University of Washington, Seattle, WA USA; 6 Department of Global Health, University of Washington, Seattle, WA USA; 7 University of Washington, Henry M. Jackson School of International Studies, Seattle, WA USA; 8 Karma Health, Kathmandu, Nepal; 9 Om Health Science Campus, Purbanchal University, Kathmandu, VA Nepal; 10 Department of Medicine, Brigham and Women’s Hospital, Division of Global Health Equity, Boston, MA USA; 11 Department of Medicine, Massachusetts General Hospital, Division of General Internal Medicine, Boston, MA USA; 12 Department of Medicine, Harvard Medical School, Boston, MA USA; 13 Yale School of Public Health, New Haven, CT USA; 14 Eastern University, College of Business and Leadership, St. Davids, PA USA; 15 Group Care Global, Silver Spring, MD USA; 16 Department of Health Systems Design and Global Health, Icahn School of Medicine at Mount Sinai, New York, NY USA; 17 Icahn School of Medicine at Mount Sinai, Arnhold Institute for Global Health, 1216 Fifth Avenue Fifth Floor, Room 556, New York, NY 10029 USA; 18 Icahn School of Medicine at Mount Sinai, Department of Pediatrics, New York, NY USA; 19 Icahn School of Medicine at Mount Sinai, Department of Internal Medicine, New York, NY USA; 20 Icahn School of Medicine at Mount Sinai, Department of Obstetrics, Gynecology and Reproductive Science, New York, NY USA; 21 Possible, New York, NY USA

**Keywords:** Group antenatal care, Group prenatal care, CenteringPregnancy, Institutional birth, Implementation science, Quality of care, Nepal

## Abstract

**Background:**

Access to high-quality antenatal care services has been shown to be beneficial for maternal and child health. In 2016, the WHO published evidence-based recommendations for antenatal care that aim to improve utilization, quality of care, and the patient experience. Prior research in Nepal has shown that a lack of social support, birth planning, and resources are barriers to accessing services in rural communities. The success of CenteringPregnancy and participatory action women’s groups suggests that group care models may both improve access to care and the quality of care delivered through women’s empowerment and the creation of social networks. We present a group antenatal care model in rural Nepal, designed and implemented by the healthcare delivery organization Nyaya Health Nepal, as well as an assessment of implementation outcomes.

**Methods:**

The study was conducted at Bayalata Hospital in Achham, Nepal, via a public private partnership between the Nepali non-profit, Nyaya Health Nepal, and the Ministry of Health and Population, with financial and technical assistance from the American non-profit, *Possible*. We implemented group antenatal care as a prospective non-randomized cluster-controlled, type I hybrid effectiveness-implementation study in six village clusters. The implementation approach allows for iterative improvement in design, making changes to improve the quality of the intervention. Assessments of implementation process and model fidelity were undertaken using a mobile checklist completed by nurse supervisors, and observation forms completed by program leadership. We evaluated data quarterly using descriptive statistics to identify trends. Qualitative interviews and team communications were analyzed through immersion crystallization to identify major themes that evolved during the implementation process.

**Results:**

A total of 141 group antenatal sessions were run during the study period. This paper reports on implementation results, whereas we analyze and present patient-level effectiveness outcomes in a complementary paper in this journal. There was high process fidelity to the model, with 85.7% (95% CI 77.1–91.5%) of visits completing all process elements, and high content fidelity, with all village clusters meeting the minimum target frequency for 80% of topics. The annual per capita cost for group antenatal care was 0.50 USD. Qualitative analysis revealed the compromise of stable gestation-matched composition of the group members in order to make the intervention feasible. Major adaptations were made in training, documentation, feedback and logistics.

**Conclusion:**

Group antenatal care provided in collaboration with local government clinics has the potential to provide accessible and high quality antenatal care to women in rural Nepal. The intervention is a feasible and affordable alternative to individual antenatal care. Our experience has shown that adaptation from prior models was important for the program to be successful in the local context within the national healthcare system.

**Trial registration:**

ClinicalTrials.gov Identifier: NCT02330887, registered 01/05/2015, retroactively registered.

## Plain English summary

Access to high-quality pregnancy care services has been shown to be beneficial for maternal and child health. Data on “group care” models, where women come together in groups and focus on their health, show they may improve both access to and quality of pregnancy care. We adapted a group pregnancy care model to the context of rural Nepal within the public healthcare system. Women were brought together four times during their pregnancy to receive care at a village clinic. During these visits, they had a check-up with a midwife and engaged in a discussion with other pregnant women and a community health worker on many aspects of having a safe and health pregnancy.

We studied the implementation of this model, looking at how we changed and improved the model and how closely we followed our program design. We ran a total of 141 group pregnancy care sessions during the study. The annual per capita cost for our model was 0.50 US dollars. We made significant adaptations to our model to make it run more smoothly, in training, documentation, feedback and logistics.

Group pregnancy care, provided by community health workers and midwives in local government clinics, can deliver accessible and high-quality antenatal care to women in rural Nepal. Group care is a feasible and affordable alternative to individual pregnancy care in this setting. Significant adaptation from prior models was necessary for the program to be successful in the local context within the national healthcare system.

## Background

Mothers and babies face extraordinary risks during childbirth. Intrapartum complications are linked to the world’s two million annual still births and neonatal deaths, and to more than 40% of the world’s 535,900 annual maternal deaths [1, 2]. Improving institutional birth rates is key to reducing maternal and neonatal mortality in low- and middle-income countries, where 99% of these deaths occur [3]. Nepal, one of Asia’s most impoverished countries, has made progress towards reducing maternal mortality. In 2015, Nepal’s maternal mortality ratio was estimated at 258 deaths per 100,000 live births, marking a 71.8% reduction compared to 1990 levels [4]. Despite these gains, Nepal is far from the new global target of less than 70 deaths per 100,000 live births and today only 57% of births take place in a healthcare facility [5].

Nyaya Health Nepal, a nonprofit healthcare organization, operates Bayalpata Hospital in a public-private partnership with the Ministry of Health and Population in Achham district in Nepal’s Far-Western Development Region with technical assistance and support from the United States-based non-profit organization *Possible*. Achham is geographically and politically isolated. Served by one major road, the hospital is approximately 12 h from the nearest tertiary care facility and domestic airport, and more than 30 h from capital city of Kathmandu by road. Communities in Achham are dispersed with an estimated population density of 153 people per square kilometer [6]. At the time of this study the Nyaya Health Nepal’s community health worker (CHW) network served a direct catchment area population of 36,000 people across 14 village clusters (known locally in Nepal as wards comprising a rural municipality). Each CHW covered a population of about 2000 and was supervised by a Nyaya Health Nepal-employed community health nurse. Each village cluster is additionally served by a government clinic, staffed by mid-level practitioners, often including nurse-midwives who are trained in skilled birth attendance.

In 2012, Bayalpata Hospital implemented comprehensive emergency obstetric care services and found that the institutional birth rate in the catchment area population significantly increased from 30 to 77% [7]. Qualitative data showed that targeting social support, birth planning, and resources may be important for reaching the remaining group of women not accessing services [7, 8]. The success of CenteringPregnancy in high-resource settings [9, 10], and participatory action women’s groups in low-resource settings [11, 12], suggested that these group care models may promote women’s empowerment and social support network development to address resource and sociocultural barriers to care. In addition, we hypothesized that the increased amount of face time with practitioners offered by the group model and decentralized high-risk pregnancy detection through prenatal labs and ultrasound, may increase birth planning success.

Secondary analysis of fidelity data for CenteringPregnancy randomized controlled trials suggests that process fidelity to the core components listed in Table 1 has a greater impact on maternal and neonatal health outcomes than content fidelity of the facilitated discussion material [14]. However, due to the significant restructuring of clinic space and time required to hold groups, adaptations that compromise process fidelity, such as reducing staff facilitators or broadening gestational age ranges in groups to increase participation, may be necessary [15]. With only a few small studies of group antenatal care (ANC) adapted from the CenteringPregnancy model for low-resource settings at the time of the design of the current intervention, in Egypt [16], Botswana [17], and one in Malawi and Tanzania [18], there was little evidence to guide adaptation, particularly in low-resource environments. We thus attempt here to provide an account of our adaptation process, as well as an assessment of implementation outcomes including fidelity, costs, and feasibility. We have published the results of an effectiveness evaluation of this intervention in a complementary paper in this journal.

**Table 1:**
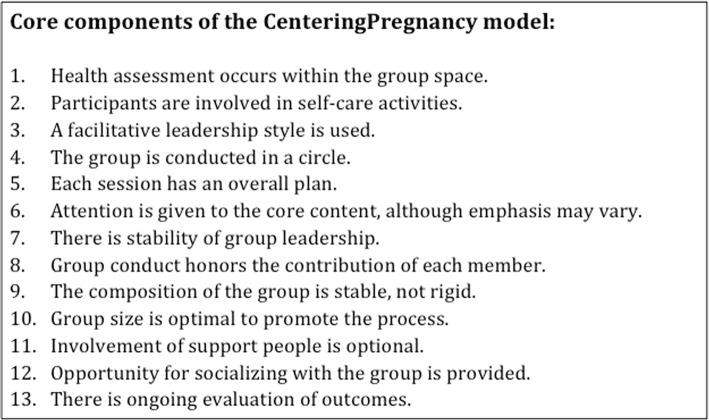
Core components of the CenteringPregnancy model, as defined by Rising, et al. [13]

## Methods

### Intervention design

The intervention draws primarily on CenteringPregnancy, a model of facilitated antenatal care groups with stable gestational-age matched composition that includes group health assessments and self-care activities, education, support and socialization, and ongoing outcomes evaluation. Table 1 shows CenteringPregnancy’s core components. CenteringPregnancy appears to improve maternal and neonatal outcomes including improved maternal satisfaction, fewer preterm births, and increased birth weight, particularly when implemented with trained and skilled facilitators [14, 19]. Group ANC has also been recognized as a health care system intervention to improve the utilization and quality of ANC by the WHO in a 2016 recommendation [20].

Additionally, we designed the intervention to extend the CenteringPregnancy model by including a participatory action process for addressing barriers to maternal healthcare access, particularly poverty and lack of resources [21]. The participatory learning and action group model takes participants through a shared process of problem identification, action, observation, and reflection [12]. Women’s groups employing this model in low-income countries including Nepal have demonstrated impact on maternal and neonatal healthcare-seeking behaviors and outcomes through increased confidence and strengthened social support [11, 22–24]. Due to this growing body of evidence, such groups are now recommended by the World Health Organization [25].

We chose to locate the groups in government village clinics to improve the quality of decentralized ANC provided at these clinics. In CenteringPregnancy, groups are led by a clinical provider while in the participatory learning and action group model, groups are led by a non-clinical lay facilitator. We designed our model to be led jointly by the government village clinic nurse-midwives and by a Nyaya Health Nepal-employed CHW. By collaboratively providing group care, we aimed to strengthen the quality of ANC, the collaboration between Nyaya Health Nepal and government staff, and the relationship between women and the village clinics.

Standard ANC at the village clinics did not include antenatal labs, except for HIV testing, or consistent antenatal ultrasound due to government resource constraints in this remote setting. Given the intervention’s focus on birth planning, and the challenge of risk-stratifying women to identify those who should deliver at a comprehensive emergency obstetric care facility, we decided to expand the scope of ANC at the village clinics during group care. Utilizing the gestational-age matched groups, we planned to provide second-trimester antenatal labs and third-trimester ultrasounds to groups of women during the appropriate time during pregnancy. Nyaya Health Nepal’s community health nurses, supervisors to the CHWs, were trained in performing point-of-care antenatal labs and basic obstetric ultrasound to identify placental location, fetal malpresentation, dating, growth, and adequacy of amniotic fluid. Women with any abnormalities identified on labs or ultrasound were referred to a higher-level facility for confirmation and management. These nurses were scheduled to provide these expanded services to women during group care at the village clinics.

The resultant intervention is called Group Antenatal Care (Group ANC). We hypothesized that this novel intervention would lead to an increased institutional birth rate by addressing the drivers of underutilization, strengthening the quality of decentralized antenatal care, and drawing on the strength within communities of women to change healthcare-seeking behaviors.

The initial design of Group ANC aimed to maintain high fidelity to all CenteringPregnancy essential components (see Table 1), but we reduced the number of visits from ten to six to align better with the government schedule for ANC incentives [26] which follows the World Health Organization’s four-visit standard [27]. The two ‘extra’ visits occurred during the seventh month and one to two months postnatal. As such, we condensed the Centering Pregnancy content and adapted to the government guidelines for ANC counseling and testing [26]. Given the short-term nature of ANC, we did not plan to fully replicate participatory action groups relying on monthly mothers’ groups that have shown success elsewhere in Nepal [22, 28]. Instead, we initially designed the model to incorporate the participatory action cycle during each visit, with participants identifying a pregnancy or childbirth-specific issue (i.e. transport availability) to address in their respective communities.

### Research design

We implemented Group ANC as a prospective non-randomized controlled, type I hybrid effectiveness-implementation study [29] in 13 village clusters employing both pre-post census and cohort questionnaires. All 13 clusters had an ‘enhanced’ standard of home-based care. The government’s Female Community Health Volunteer program offers limited ANC counseling and distribution of iron and folic acid tablets to pregnant women. In all 13 clusters in this study, Nyaya Health Nepal implemented a CHW program, assigning a local, literate, and trained CHW to a geography covering about 2000 people. CHWs visited all pregnant women monthly to conduct trimester-specific counseling and birth planning, using the same written materials as used in the Group ANC sessions. The six intervention clusters had Group ANC implemented at village clinics in addition to the CHW home visit program. The intervention was offered to all known pregnant women living in the six intervention village clusters, either at the time of presentation to the clinic or during routine home-based surveillance by CHWs. The seven control clusters continued with the current standard of facility-based antenatal care with individual clinic visits.

We measured primary population-level outcomes, including institutional birth rate and antenatal care completion, through a comparison of a household census conducted in the catchment area population prior to implementation of the intervention and after one full year of implementation. We also measured secondary intervention individual-level outcomes (changes in knowledge, attitudes, and birth planning behavior) by a pre-post questionnaire administered to a nested cohort; these are reported in a complementary paper in this journal.

We undertook ongoing assessments of implementation process and model fidelity, from May 2015 to April 2016, using a mobile checklist developed using SurveyCTO [30], a mobile data collection platform for Android phones, which nurse supervisors filled out during every visit. We measured process fidelity primarily in three ways: (1) as the proportion of visits that fulfilled all process checkpoints; (2) the proportion of visits in which all women were engaged and supportive of one another; and (3) the proportion of visits with a score of four or five on the didactic versus peer-to-peer counseling scale. We assessed content fidelity in two ways: (1) the relative frequency of topics (quarterly and overall) compared to the expected frequency based on counseling guidelines, and (2) the percentage of visits (quarterly and overall) in which documented topics matched the visit-specific counseling guidelines. The matched data are only available during the last two quarters, as they were documented during the first two quarters.

We evaluated these quantitative data quarterly using descriptive statistics to identify benchmarks and trends. We used JMP software Version 11 (JMP®, Version 11. SAS Institute Inc., Cary, NC, 1989–2007) and SAS software, Version 9.3 of the SAS System for Microsoft (SAS Institute Inc., Cary, NC, USA) for data cleaning and analysis.

Program leaders also completed semi-structured group observation forms once a month in each village cluster, which we analyzed recursively during the study to guide adaptation. Piloting and iteration of the group intervention was conducted from September 2014 to February 2015. At the end of the pilot, we completed one focus group discussion with nurse-midwives. At endline, we undertook three key informant interviews with program leadership and collated team communications on Asana, a project management platform that Nyaya Health Nepal uses in lieu of email, meeting minutes, and memos. Together these data were analyzed through immersion crystallization [31] to identify major themes that evolved during the intervention adaptation and implementation process.

## Results

141 Group ANC sessions were conducted in total across six village clusters during the study period, from May 2015 to April 2016. This included 41 groups for 4th and 6th month women, 36 groups for 8th and 9th month women, 44 groups for all gestational ages, and 20 postnatal groups. The median attendance per session was 8 participants [IQR: 4,12], excluding 13 sessions that were missing data on attendance.

### Fidelity

We aimed to maintain fidelity to as many of CenteringPregnancy’s core components as possible, in both process and content (see Table 1). The data for percentage of visits meeting all process checkpoints (including a planning meeting, introductions, sitting in a circle, active participation in exams, documentation, and closing) were only available after the first quarter due to changes in the supervisory checklist. The available data indicated consistently high process fidelity in these areas, with 85.7% (95% CI 77.1–91.5%) of visits completing all six elements. All elements were completed more than 99% of the time, except for physical exams for all women, which were completed during 86.8% of the visits. Half of the visits in which physical exams for all women were not completed were postnatal visits (6/12); postnatal physical exams were not part of routine care provided by government nurse-midwives. The other visits were either “mixed” group visits, with no gestational-age matching, or larger groups (11 to 19 women).

Based on observation, physical exams were often the most challenging component to organize, requiring significant rearrangements of clinic space as well as a high degree of coordination to execute efficiently. Another major stumbling block was documentation, as much time was spent determining whether a woman was eligible for Safe Motherhood incentives, which determined whether her visit would be documented on her government ANC card. If a woman was deemed ineligible, her physical exam was occasionally neglected. Overall, however, women in groups received more thorough examinations than they would during a standard clinic visit due to the availability of prenatal labs and ultrasound. Assuming a total of 300 participants, we estimate that 82% (247) received prenatal labs and 85% (255) received third trimester ultrasound. We are unable to speculate on those participants who may have received those services at Bayalpata Hospital or elsewhere. Through the diagnostic testing during Group ANC sessions, we identified 53 high-risk cases. We estimate this to be approximately 18% of total participants.

Group dynamics were another core component of process fidelity. As assessed by the percentage of visits in which most or all women were actively engaged (93.6, 95% CI 88.3–96.6%), sharing (68.0, 95% CI 60.0–75.2%), supportive (69.5, 95% CI 61.5–76.5%) and not distracting (88, 95% CI 82–92%), participant dynamics were good overall. Nominal logistic regression analysis measuring village cluster effect and time in quarters (and cluster-time) indicated significant improvements over time for the “supportive” and “not distracting” ratings (both *p* < 0.01) and a positive but non-significant time effect in the “sharing” and “actively engaged” categories (0.28 and 1, respectively) as shown in Table 2. We did find a significant difference across village clusters in the sharing category (*p* = 0.03), with two clusters rating “most to all” 40.9% (95% CI 23.2–61.3%) and 52.3% (95% CI 32.4–71.7%) of the time, compared to the other clusters (77.6, 95% CI 68.3–84.7%).

**Table 2:** Group dynamics assessments over time

Group Dynamics	Quarter 1	Quarter 2	Quarter 3	Quarter 4	Overall	*P*-value^a^
	(%, 95 CI)	(%, 95 CI)	(%, 95 CI)	(%, 95 CI)	(%, 95 CI)	
Number of groups	49	26	28	38	141	–
Most to all engaged	81.6% (68.6–90.0%)	100.0% (87.1–100.0%)	100.0% (87.9–100.0%)	100.0% (90.8–100.0%)	93.6% (88.3–96.6%)	< 0.01
Most to all sharing	61.2% (47.2–73.6%)	61.5% (42.5–77.6%)	71.4% (52.9–87.4%)	78.9% (63.7–88.9%)	68.1% (60.0–75.2%)	0.28
Most to all supportive	44.9% (31.9–58.7%)	84.6% (66.4–93.9%)	82.1% (64.4–92.1%)	81.6% (66.6–90.8%)	69.5%(61.5–76.5%)	< 0.01
Most to all not distracting	65.3% (51.3–77.1%)	100.0% (87.1–100.0%)	100.0% (87.9–100.0%)	100.0% (90.8–100.1%)	87.9% (81.5–92.3%)	< 0.01
Peer group rating 4 or 5	77.6% (64.1–87.0%)	84.6% (66.5–93.9%)	89.3% (72.8–96.2%)	78.9% (63.7–88.9%)	81.6% (74.4–87.1%)	0.55

^a^*P*-values calculated via nominal logistic regression analysis by quarter

Data are percentages of all reported visits in all village clusters, by quarter and over 1 year (May 2015 to April 2016)

Similarly, the percentage of visits with a strong peer-group facilitation rating was consistently above 75% (median score of four quarterly average of 77–89% visits scoring four or five, see also Table 2), except in one village cluster in which only 59% of all visits rated above three. Interestingly, this village cluster (H) and the cluster with the lowest “sharing” ratings (S) had both been identified as having weak nurse-midwife engagement, and were groups fully “mixed” by gestational age. Anecdotally, the mixed group sessions were more challenging because of the different topics of interest to women in early and late pregnancy.

With respect to content fidelity, we found topics varied in relative frequency, though the quarterly average of all village clusters for each topic met the minimum target frequency except parenting and newborn danger signs (32, 95% CI 21–42% versus an expected 40%). Pregnancy danger signs (70, 95% CI 62–78%), nutrition (51, 95% CI 39–64%), and contraception (42, 95% CI 53–31%) were the most frequent, which compared favorably with the expected frequencies of 40, 20, and 20% respectively. The least frequent topics were postdates induction (21, 95% CI 14–29%) and relaxation techniques (26, 95% CI 17–35%), all of which had an expected frequency of 20%. The relative frequency of each topic also varied by village cluster, as shown in Table 3.

**Table 3:** Quarterly frequency of each topic discussed

Discussion Topic (target %)^a^	Village Cluster^b^						
	B	H	J	L	P	S	All
Nutrition (20%)	40%	56%	39%	62%	56%	57%	51%
Self-Esteem (20%)	33%	43%	***17%***	24%	51%	24%	32%
Sexuality in pregnancy (20%)	44%	40%	34%	37%	51%	30%	39%
Gender based violence and abuse (20%)	22%	25%	35%	34%	33%	35%	31%
Pregnancy danger signs (40%)	69%	72%	66%	78%	49%	85%	70%
Postpartum contraception (40%)	52%	***35%***	***30%***	49%	55%	***33%***	43%
Parenting/newborn danger signs (40%)	***36%***	***31%***	***30%***	***37%***	***31%***	***29%***	***32%***
Birth stories (20%)	39%	31%	36%	29%	33%	31%	33%
Relaxation techniques (20%)	44%	33%	25%	***10%***	32%	***15%***	26%
Postdates induction (20%)	17%	29%	***15%***	***13%***	***16%***	39%	21%
Birth Plan: finances (25%)	49%	37%	***19%***	***16%***	33%	31%	31%
Birth Plan: support (25%)	***16%***	48%	65%	28%	31%	36%	37%
Birth Plan: transportation (25%)	35%	***15%***	***16%***	56%	35%	33%	32%

^a^Average relative frequencies below the respective topics’ target are highlighted in bold and italics

Village clusters (all located in Achham, Nepal in Bayalpata Hospital catchment area): B=Bardadevi, H=Hattikot, J = Jalpadevi, L = Lungra, P=Payal, S=Siddheswor

Guideline fidelity, as measured by percentage of visits that followed specific topic guidelines, was 66% (95% CI 54–77%). Providers were given an opportunity to state their planned topics (regardless of the guidelines) and followed their plan about 77% (95% CI 68–86%) of the time. Together these data suggest that providers used the guidelines somewhat flexibly but adequately covered counseling topics over the five-visit cycle.

Birth planning content fidelity was slightly less successful. For birth planning, providers were instructed to cover a rotating topic (e.g. finances, support, or transportation) over the course of four antenatal visits, with women completing a worksheet for their specific birth plan. Thus, the minimum relative frequency was 25% for each of these topics, a target met on average 66.7, 66.7, and 62% of the time, respectively. Quarterly averages by village cluster for birth planning topic relative frequencies are also presented in Table 3.

### Costs

The annual per capita costs for Group ANC were marginal (0.50 USD) compared to Nyaya Health Nepal’s goal of 25 USD. The initial three-day trainings and two-day re-trainings of supervisors, CHWs, and nurse-midwives cost an estimated total of 92,750 Nepali Rupees (NRS, about 861 USD). Excluding training and including CHW time, nurse supervisor time, portable ultrasounds, and lab supplies, the intervention had an estimated average monthly cost of approximately 97,400 NRs (904 USD), an average per-visit cost of 8119 NRs (75 USD), an average per-woman cost of 4000 NRs (37 USD, per woman completing four visits), and annual per capita cost of 0.50 USD across the 23,000 person intervention cluster catchment area population. The final costs are less than half the estimated pilot monthly cost of 198,500 NRs (1843 USD), during which there were four less CHWs but 50% more monthly visits, as well as a lab technician and ultrasound-trained midwife to supervise diagnostics.

Notably, pregnant women typically received 10-min individual visits with an average of 1 min for ANC counseling at village clinics [32]. By comparison, a 120-min group session of 12 women was essentially equivalent in time spent for the nurse-midwife while each woman received at least 90 min of counseling (estimating an average of 30 min for physical exams). With that in mind, Group ANC was likely to be relatively cost-effective even when including the added costs of Nyaya Health Nepal’s facilitation and diagnostic support.

### Feasibility

We sacrificed some fidelity to the CenteringPregnancy component of group member stability in favor of a simplified scheduling system, which also increased compatibility with government protocols and organizational workflows. This change dramatically improved the feasibility of the intervention. The fixed scheduling made the model “more intuitive to the government health workers and thus made them more comfortable in implementing” (non-clinical research staff member, KII, January 28, 2016). In addition, through this process, the team made,

Tremendous strides in building the rapport relationship with the (District Health Office) and they have had to put a lot of effort into that -- just as much effort into that as into the real thinking behind what the components of the program look like -- like how do you build a relationship with the government at the district level. (program leader, KII, December 7, 2015).

Considering the cost of the current model and Nepal’s current per capita health expenditures of 10 USD, Group ANC was feasible for Nyaya Health Nepal due to external grants but would not be feasible for scale on a national level unless significantly restructured. It should be noted that the government is considering professionalizing its current CHW program. This strategy could potentially improve viability of the program as the major associated cost is Nyaya Health Nepal’s CHW and supervisor time. Professionalized government CHWs could conduct home visits to complement facility-based ANC and birth planning, as well as support government nurse-midwives for group visits at the village clinics. Alternatively, with the adapted scheduling system, government nurse-midwives could easily coordinate and manage group visits alone without the support of professional CHWs, especially if government Female Community Health Volunteers were given increased training and incentives to participate.

Currently, the only access that most rural women have to obstetric ultrasound services comes from a camp-style government ultrasound program. These services are not regularly scheduled and women may not have access to an ultrasound at the appropriate time during pregnancy. Group ANC provides a feasible model for increasing ANC services at the village clinic level to include labs and ultrasounds, as women of the appropriate gestational age are gathered from the village cluster at one time to receive care. Skilled staff and equipment would need to be provided on a monthly basis from a higher-level facility, in order for the costs to be shared over a larger geography.

## Discussion

During the seven months of piloting, a number of major issues were highlighted, several similar to previously cited CenteringPregnancy implementation barriers [14, 15, 33]. These issues included scheduling challenges and unpredictable group attendance, difficulty engaging nurse-midwives in the practice, overly didactic group discussions, and sub-standard documentation. Attempting to combine participatory learning and action cycles with antenatal care proved to be infeasible. In addition, the costs associated with bringing ultrasound-trained nurse-midwives and lab technicians from Bayalpata Hospital were found to be unsustainable. In the following sections, we review these problems, our implementation strategies, and intervention adaptations.

### Scheduling and group stability

The Nepal National Safe Motherhood Program provides a financial incentive to women who complete four ANC visits, but only if those visits are completed during the fourth, sixth, eighth, and ninth month of gestation [26]. The government tracks ANC coverage by the percentage of expected pregnant women that fulfill these requirements, and rewards clinics with the highest rates. Like the CenteringPregnancy model, the groups were initially composed of women who would deliver in the same month. This model created conflict with the government policy for two reasons: (1) the visit schedule for gestational month-matched groups sometimes led participating women to miss the strict dates of attendance that the government required to provide the financial incentive, and (2) inconsistent gestational age calculations between providers meant that even if schedulers attempted to ensure women met all government attendance dates, they would ultimately be deemed ineligible for the incentive.

According to clinical providers, the gestational month groups helped focus the discussions with women, but the scheduling challenges felt too chaotic and ultimately left some women without incentives, and clinics with worsened coverage numbers. They suggested fixed monthly dates instead (focus group discussion, December 2015). Government officials were unable to allow more flexible incentive eligibility policies that would have accommodated both stable gestational-age-group scheduling and fixed monthly visits.

In the adapted model, group sessions were held bi-weekly and women attended as needed to meet the government-defined eligibility windows. This adaptation loosened CenteringPregnancy’s requirement for stable groups but maintained some gestational-age focus by separating these drop-in groups into a second trimester (fourth to sixth month) and a third trimester (eighth to ninth) group each month. In addition, there were two village clusters in which gestational-age based visits were not feasible simply due to small population sizes, resulting in fewer pregnant women at any given time. These two clusters received “mixed group” counseling guides that rotated through the same content as the modified gestational-age based groups.

### Nurse-midwife engagement

One of the key issues identified during piloting centered on how government-employed nurse-midwives engaged in the Group ANC program. While Nyaya Health Nepal-employed CHWs and nurse supervisors were asked to defer counseling and examination to nurse-midwives due to their role as government ANC providers, initial observations suggested that often nurse-midwives were either distracted by other clinic duties or relied heavily on didactic facilitation techniques. Conflict with their other responsibilities was due in part to scheduling issues addressed above and was addressed accordingly. Given that Nyaya Health Nepal has no management of these government-employed nurse-midwives, it was necessary to heavily engage the leadership of each clinic as to provide adequate support and oversight. While improving over time, this process remained challenging. Understanding that, as lay providers, CHWs may have difficulty giving feedback to nurse-midwives, we offered a re-training for both CHWs and nurse-midwives and began tracking nurse-midwife attendance, and level of engagement in counseling and examinations. We also asked nurse supervisors to help lead a pre-session planning meeting and post-session debriefing that included both the CHWs and the government nurse-midwives.

### Facilitation quality

We observed highly didactic facilitation in the initial pilot sessions, with nurse-midwives and CHWs often reading from the guidelines and few women participating in discussion. Facilitators expressed difficulty drawing out women, who were often shy and unused to speaking in more formal environments such as a village clinic. As one clinical provider reported, “in the beginning they were unable to even say their names. They told us, ‘I can’t, I won’t’” (key informant interview, December 2015).

In addition, nurse-midwives had years and sometimes decades of experience providing one-on-one ANC. Like many healthcare providers, including those in vastly different settings, their traditional style was often to impart pertinent medical information without adequate consideration of a patient’s prior understanding, social context, or beliefs. To facilitate a group discussion that encourages peer-to-peer sharing of experiences and information, while ensuring that evidence-based information is understood, is thus a great departure from providers’ traditional experience.

We worked to improve this facilitation quality through a two-day training at the end of the pilot that emphasized the peer-group model through role-play. During consultation with organizers of other women’s groups in rural Nepal, our nurse supervisors learned various games and strategies useful when women resist open discussion, and these were also directly added to the guides and trainings. Finally, we encouraged routine and “real-time” feedback from nurse supervisors during the post-session debriefings through the immediate use of the checklist data.

### Documentation

Visit documentation was a significant challenge on the part of both CHWs and nurse-midwives. CHWs are responsible for keeping a registry of all pregnant women in their catchment area, which initially included data on last menstrual period, completed visit dates, risk factors, and birth outcomes. Nurse-midwives maintain a government registry that includes the same information as well as more specific clinical documentation. Our aim was to ensure that both registries had, at a minimum, the same women and the same visits, but we struggled to find an adequate system of reconciliation. As one program leader noted at the end of the pilot:

We still do not have a strong data collection system that fits into the workflow. Data were not collected because no one entered them into the government registry. The CHWs wrote the information on a piece of paper and said they would try and get it to the village clinic auxiliary nurse midwife tomorrow or the day after so that the information could be entered into the government registry. CHWs said they did not have time to properly record in their registries and would do it later. They did not want to make errors on their paper registry forms (which I understood because it would have been hard to enter the data later) so they wrote the patient information on a piece of paper quickly and would enter properly later. (Asana, May 2015).

We attempted to simplify the CHW’s registry by removing visit date fields, instead organizing visits by eligibility windows that matched the government’s defined schedule. CHWs found this registry easier to use, but required continuous supervision to ensure completeness and accuracy. Unfortunately, data quality concerns prohibited us from relying on these registries for this current evaluation. As a result, over the course of the study year we began adapting a different mobile application CommCare, with the mobile health company Dimagi [34], that CHWs can use to track pregnancies and support antenatal counseling at home or in group visits. In addition, this application integrated with the hospital’s electronic health record system to improve care coordination for referrals and reduce duplicate documentation. The application was implemented in May 2016 and has continued to be improved based on user feedback.

### Participatory action and birth planning

Despite consultation with another Nepali organization experienced in participatory women’s groups, we struggled to fully implement the participatory action component of the model due to the limited number of visits, the loss of group member stability, and the significant number of other topics to address during each visit. Instead, group problem solving took place through collaborative discussion of birth plans for each woman. The fidelity data show that even these discussions were not comprehensive, as the content target for birth planning was only achieved about two-thirds of the time. Much of the individualized birth planning was transitioned to take place during CHW home visits, similar to how it was being done in the control clusters. This also allowed for family involvement in discussions and privacy regarding resources and finances.

### Ultrasound and lab services

During the pilot, the standard staff responsible for ultrasound and lab services at the hospital were asked to travel to the village clinics to provide these services remotely several times per month. The cost of this decentralized care included missed time providing care at the hospital and the utilization of specialized staff. This cost was recognized as quite high, and a plan for task shifting was made. After the pilot, nurse supervisors received training from hospital staff to complete basic prenatal labs and ultrasound themselves. One-day lab training was designed at the hospital for nurses to learn methods and run several tests for patients presenting to the hospital. Alongside this training, we developed protocols for testing and disclosing results for the nurses. Similarly, we designed a two-day ultrasound training cover basic obstetric ultrasound. This was followed by several two-hour ‘practical’ sessions with the nurse-midwives at the hospital conducting ultrasounds in the high-volume hospital outpatient department. Nurses needed to complete a competency assessment before conducting ultrasounds in the village clinics. During the initial weeks of decentralized service delivery, hospital staff accompanied the nurses to provide supervision, assess quality, and provide feedback.

### Study limitations

Weaknesses in routine documentation limited both our implementation and our evaluation process. The absence of complete patient records makes quantification of coverage less reliable. We worked to improve documentation through simplification of CHW forms and changes to the nurse supervisor forms, but were unable to integrate with or improve village clinic documentation.

In addition, social desirability bias and concerns over performance evaluations may have contributed to routinely high fidelity data, particularly with respect to the more subjective elements regarding group dynamics and facilitation quality. Direct observations by program leadership and research staff tended to reveal more weaknesses in these areas than the checklists suggest. We attempted to increase the impartiality of the checklist data through repeated supervisor trainings; in addition, there were no Group ANC-specific performance incentives in place during the study.

Finally, our experience implementing Group ANC is in many ways highly context-specific and influenced by the particular resources, historical relationships, and culture of the organization and community. Nonetheless, we believe similar issues are likely to be present in low-resource settings with dispersed patient communities and centralized government policies and incentives for ANC.

## Conclusions

As others have, we faced challenges implementing Group ANC. Group care requires significant shifts from traditional practice, including revised staffing and scheduling systems, and a transition from a didactic clinical model to a facilitated peer-group experience that elevates women’s collective knowledge and encourages greater participation in care. Due to Nyaya Health Nepal’s commitment to strengthening the public healthcare system and the dependency of Group ANC on the participation of village clinic nurse-midwives, scheduling in accordance with government policy was a priority that resulted in significant adaptations of the initial gestational age-based design. Additionally, we iterated upon the guidelines and facilitator trainings to emphasize the importance of peer group discussions.

Overall, the adapted Group ANC model maintains a focus on creating a supportive, empowering atmosphere and providing high-quality counseling and basic diagnostics. Observations during a pre-study pilot period indicated that fidelity to key elements of the CenteringPregnancy process, namely those that facilitate peer-discussion (rather than didactic counseling), was low. After re-training, streamlining of the guidelines along with scheduling adaptations, and increasing immediate feedback to facilitators, routine checklist data and semi-structured observations suggest that fidelity to these aspects of the CenteringPregnancy process was high. Group stability remains a concern given that women attend on a “drop-in” basis according to their specific ANC windows. We continue to evaluate and iterate on the model in each of our village clusters based upon data, observations, and input from our government partners.

Intervention feasibility vastly improved after adaptation of the scheduling scheme, which simplified Nyaya Health Nepal’s administrative workflows, and garnered greater buy-in from government officials and local healthcare providers. Additionally, decentralized antenatal lab and ultrasound service feasibility greatly improved with task-shifting to the CHW nurse supervisors. However, affordability is a concern and will likely impact sustainability unless Group ANC can be fully transitioned to government providers or unless the government creates a professional CHW cadre. Until that time, Nyaya Health Nepal will continue to offer Group ANC in collaboration with the government clinics and is currently expanding the program to other areas of its catchment area population.

We have demonstrated that Group ANC provided in collaboration with local government clinics is a potentially feasible and affordable alternative to individual ANC. As expected, the model required some adaptation from the CenteringPregnancy and participatory action women’s group models to fit into the local context and national healthcare system. However, we have shown that the current model was implemented with fidelity to the major components of CenteringPregnancy. We hope that our experiences here will be useful to others planning similar programs in underserved communities in Nepal and around the world.

## Data Availability

De-identified quantitative data are available on request by emailing: research@possiblehealth.org and will be posted in a publicly-accessible data repository. Full transcripts of qualitative data are not available as they contain quotes and identifiable information that could compromise the identify of participants.

## Abbreviations

ANC: antenatal care
CHW(s): community health worker(s)
CI: confidence interval
NRs: Nepali Rupees
REDCap: Research Electronic Data Capture
USD: United States dollar
